# Cutaneous Toxicities of Advanced Treatment for Cutaneous Melanoma: A Prospective Study from a Single-Center Institution

**DOI:** 10.3390/cancers16213679

**Published:** 2024-10-30

**Authors:** Federico Venturi, Giulia Veronesi, Biagio Scotti, Emi Dika

**Affiliations:** 1 Oncologic Dermatology Unit, IRCCS Azienda Ospedaliero-Universitaria di Bologna, 40138 Bologna, Italyemi.dika3@unibo.it (E.D.); 2 Department of Medical and Surgical Sciences (DIMEC), Alma Mater Studiorum of Bologna, 40126 Bologna, Italy

**Keywords:** immunotherapy, melanoma, toxicity, ipilimumab, nivolumab, pembrolizumab, cutaneous toxicity

## Abstract

Background/Objectives: The landscape of advanced melanoma treatments has shifted dramatically in recent years. Target therapy and immunotherapy have changed the management of patients with both metastatic (stage IV according to AJCC 8th ed.) and nodal (stage IIB/C and III) disease. As the use of novel agents has increased, so have the cutaneous toxicities associated with these medications. While most skin reactions are low-grade and can be managed conservatively with topical therapies, high-grade or life-threatening drug reactions can arise during therapy, requiring prompt dermatologic recognition and treatment. Given the survival benefit attributed to these new agents, treating skin toxicity and maintaining a patient’s quality of life is of paramount importance. Methods: We undertook a prospective, monocentric, and descriptive study in Bologna, Italy, including patients referred to the Oncologic Dermatology Unit of IRCCS AOU of Bologna who developed biopsy-proven cutaneous adverse events (AE) under treatment with immunotherapy for cutaneous melanoma with nodal (stage IIB/C, III) and metastatic (stage IV) disease from January 2016 to April 2024. Results: In 202 identified patients, 75 (37.5%) developed skin AEs. Ipilimumab was causal for 48.1% of skin AEs, followed by nivolumab (37%) and pembrolizumab (31.4%). Recorded types of skin AEs included erythematous rash, vitiligo, alopecia, lichenoid, maculopapular, acneiform, urticarial, psoriasiform, granulomatous, eczematous, and severe cutaneous AEs, such as Erythema multiforme/Stevens-Johnson syndrome and bullous autoimmune dermatoses. Most AEs were low-grade [CTCAE 1–2] (97%) and typically occurred after 10 weeks of treatment. Conclusions: This study comprehensively describes skin AEs occurring during systemic treatment with ICIs for cutaneous melanoma at a single center.

## Simple Summary

Cutaneous irAEs are some of the most reported adverse reactions to ICIs. This prospective monocentric study seeks to offer a comprehensive overview of the skin toxicity associated with these therapies in a single-center institution, with the aim of describing their incidence and our approach for management. Additionally, we aim to highlight the importance of dermatological assessment for affected patients, as it can have a major impact on both their quality of life and the decision to continue treatment.

## 1. Introduction

Cutaneous melanoma (CM) is a malignant tumor, and its incidence has increased globally in recent years [1,2,3,4]. Although CM accounts for only 3–5% of all skin cancers, it determines approximately 65% of all skin cancer deaths. Immune checkpoint inhibitors (ICIs) have revolutionized the treatment of advanced cutaneous melanoma, offering significant improvements in survival and disease control [5,6,7,8]. These therapies, which include anti-CTLA-4 (ipilimumab) and anti-PD-1/PD-L1 antibodies (nivolumab, pembrolizumab), work by unleashing the immune system to recognize and attack tumor cells. However, the same mechanisms that enhance anti-tumor immunity also predispose patients to immune-related adverse events (irAEs), with cutaneous toxicities being among the most common [9,10,11,12,13].

Cutaneous melanoma is highly immunogenic, making it particularly responsive to ICIs. These drugs block inhibitory signals that prevent immune cells from attacking cancer, thereby activating T-cells and enhancing immune responses [14,15,16,17]. While this immune reactivation benefits tumor control, it can also lead to autoimmunity against normal tissues [18,19]. Particularly, the skin is often the first site of irAEs, and cutaneous toxicities can range from mild rashes to severe, life-threatening conditions such as Stevens-Johnson syndrome or toxic epidermal necrolysis. Common cutaneous manifestations include maculopapular rashes, pruritus, vitiligo, lichenoid dermatitis, and eczema. These side effects not only affect the patient’s quality of life but can also necessitate interruption or discontinuation of cancer therapy in severe cases [11,20,21,22,23,24,25,26,27].

Understanding the mechanisms behind these toxicities, their clinical presentations, and management strategies is essential for optimizing treatment outcomes. Early identification and appropriate management of cutaneous irAEs are critical to balancing effective melanoma management and minimizing the impact on the patient’s well-being [10]. This prospective monocentric study explores the range of cutaneous toxicities associated with ICIs in cutaneous melanoma patients referred to the Oncologic Dermatology Unit of the IRCCS Azienda Ospedaliero-Universitaria of Bologna, Policlinico of Sant’Orsola, from January 2016 to April 2024.

## 2. Materials and Methods

We undertook a prospective, monocentric, and descriptive study in Bologna, Italy. Patients that were treated with immunotherapy for cutaneous melanoma with nodal (stage IIB/C, III) and metastatic (stage IV) disease and referred to the Oncologic Dermatology Unit of the IRCCS Azienda Ospedaliero-Universitaria of Bologna, Policlinico of Sant’Orsola, for cutaneous toxicities from January 2016 to April 2024 were included. This study was approved by our local Ethics Committee. Epidemiological, efficacy, and safety data were collected. The safety evaluation was performed upon the data of reported AEs according to the Common Terminology Criteria for Adverse Events v. 5.0 (CTCAE) classification and severity assessment [28].

After verifying their completeness, conformity, and plausibility, these data were subjected to specific statistical analyses. Specifically, the univariate and multivariate analysis of the factors potentially impacting OS was carried out using the Cox linear regression model. As regards the prognostic data, with a view to minimizing the influence exerted on the survival curves by the latency time between the onset of ICIs and the onset of cutaneous irAEs, a Landmark analysis was carried out 3 months from the start of ICIs, including only patients who were alive at that point.

These patients were then divided into two groups: patients who had been subject to skin toxicity within the first 3 months of therapy vs. patients who had not been subject to it. The analysis of the overall survival (OS) of the two groups was carried out using the Kaplan–Meier method.

## 3. Results

In 202 identified patients, 75 (37.5%) developed skin AEs. Among ICIs, ipilimumab was causal for 48.1% of skin AEs, followed by pembrolizumab (31.4%) and nivolumab (37%). Recorded types of skin AEs included erythematous rash, vitiligo, alopecia, lichenoid, maculopapular, acneiform, urticarial, psoriasiform, granulomatous, eczematous, and severe cutaneous AEs, such as exfoliative dermatitis and bullous autoimmune dermatoses. Most AEs were low-grade [CTCAE 1–2] (93%) and typically occurred after 10 weeks of treatment. A total of 25.3% of patients affected by skin toxicity presented two or more subsequent episodes, for a total of 104 episodes observed. In particular, the recurrence of multiple cutaneous irAEs per individual patient was observed predominantly following treatment with PD-1 inhibitors (nivolumab, 35%; pembrolizumab, 18.8%) compared to treatment with ipilimumab (15.4%). Data regarding our study cohort are fully displayed in Supplementary Table S1.

### 3.1. Incidence of Cutaneous irAEs

**Figure 1:**
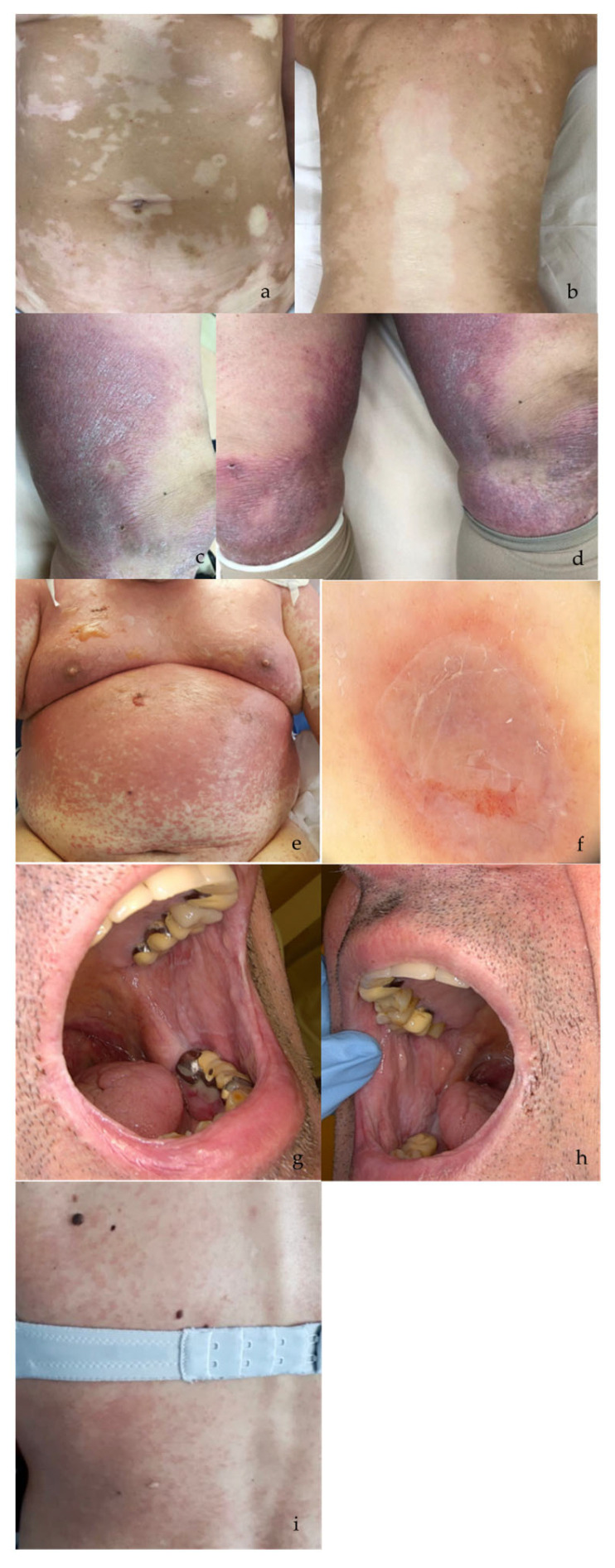
Cutaneous toxicities related to ICIs in melanoma patients: Vitiligo irAE in a 58-year-old patient with metastatic melanoma (stage IV) treated with anti-PD-1 antibody (nivolumab) (**a**,**b**). Flare-up of psoriasis in an 83-year-old patient with metastatic melanoma (stage IV) treated with anti-PD-1 antibody (pembrolizumab) (**c**,**d**). Exfoliative dermatitis (NET syndrome) in a 67-year-old patient with stage III melanoma treated with anti-PD-1 antibody (nivolumab). Clinical (**e**) and dermoscopic (**f**) presentation of disease. Erosive lichenoid reaction in an 80-year-old patient with metastatic melanoma (stage IV) treated with anti-PD-1 antibody (pembrolizumab) (**g**,**h**). Erythematous rash in a 46-year-old patient with stage IIc melanoma treated with anti-PD-1 antibody (nivolumab) (**i**).

**Figure 2:**
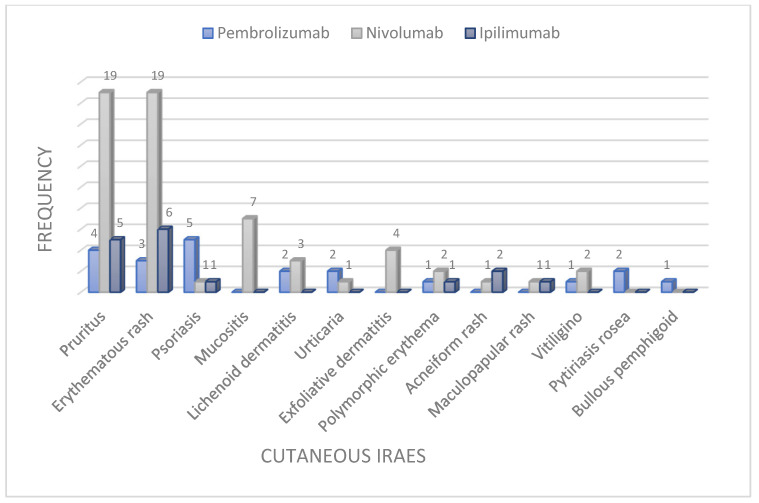
Graphical representation of cutaneous irAEs in our study population with distinction for each ICIs agent (pembrolizumab, nivolumab, ipilimumab, respectively).

The most common irAEs were pruritus (28.8%) and erythematous rash (27.9), followed by three manifestations, each found with an incidence of 7%: psoriasis (flare-up or first appearance), mucositis, and lichenoid dermatitis. Finally, more rarely found irAEs were urticarial rash, exfoliative dermatitis, polymorphic erythema, acneiform rash, maculovesicular rash, vitiligo, pityriasis rosea, alopecia, and bullous pemphigoid (Figure 1a–i and Figure 2).

**Figure 3:**
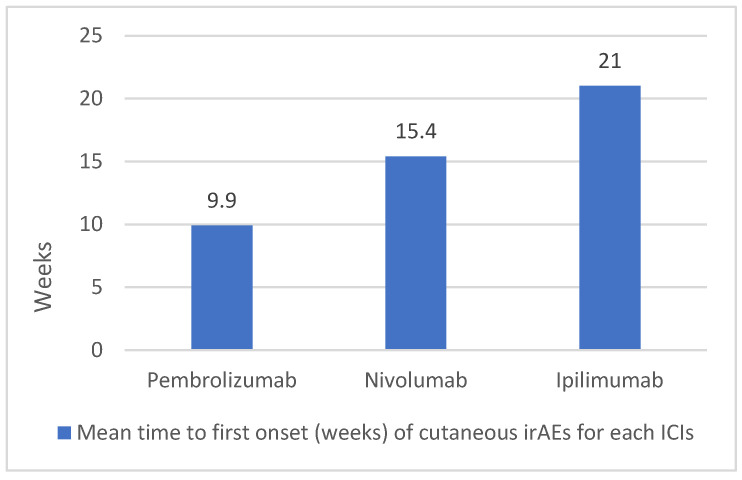
Graphical representation of mean time to first onset (weeks) of cutaneous irAEs in our study population.

**Figure 4:**
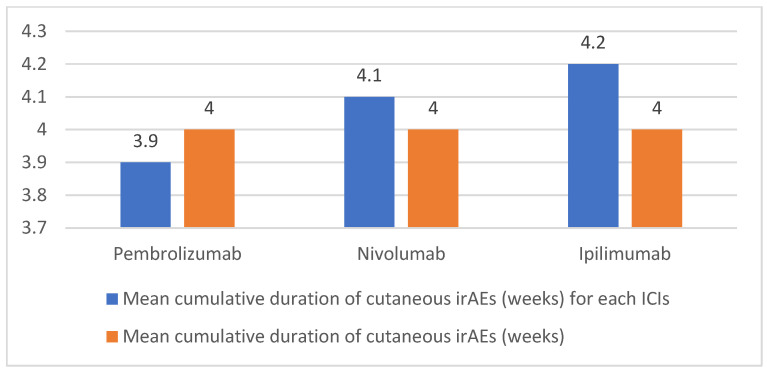
Graphical representation of mean cumulative duration (weeks) of cutaneous irAEs in our study population.

The median time to onset was typically after an interval of 10 weeks. In particular, the onset time was shorter in the case of PD-1/PD-L1 inhibitors (approximately 10–17 weeks) compared to ipilimumab (21 weeks) (Figure 3). From the analysis of the data, dermatological irAEs from ICIs last on average for 4 weeks; nevertheless, it is important to observe the presence of considerable heterogeneity in the duration and course of these events (Figure 4).

In fact, toxicities that resolve within a few days and toxicities that last for 1–2 years have been found. In this last case, these are mild toxicities, partially controlled with symptomatic therapies, such as topical emollients or antihistamines, but which can also persist for the entire duration of the ICIs treatment.

As regards the severe toxicities found, the duration did not exceed 12 weeks.

No relevant differences were found in the average duration of cutaneous irAEs of the individual immunotherapy drugs, the medians differing by only a few days.

### 3.2. Severity of irAEs

The observed cutaneous irAEs were, in most cases, mild (Grade 1 according to the CTCAE v. 5 classification) or moderate (Grade 2): 57% and 36%, respectively, for a total of 93% of mild–moderate toxicity.

**Figure 5:**
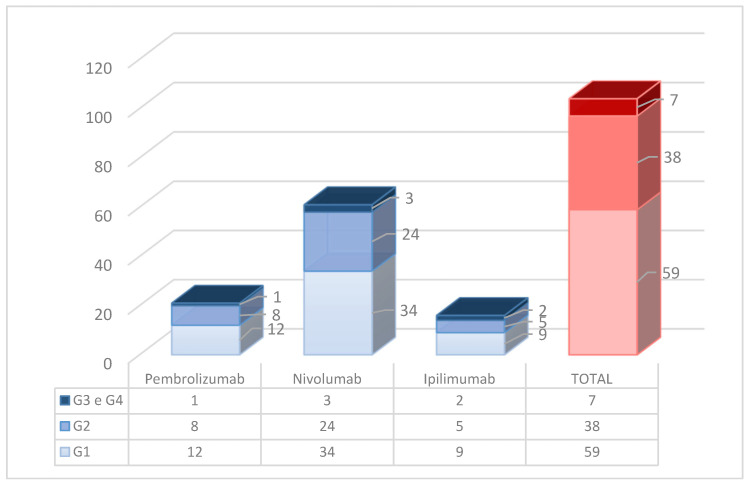
Graphical representation of severity of cutaneous irAEs in our study population (according to CTCAE v 5.0).

Nonetheless, severe toxicities (Grade 3 or 4) were observed in a limited number of patients, requiring suspension (temporary or permanent) of immunotherapy; overall, severe dermatological irAEs accounted for 7% of the total (Figure 5).

Regarding severe toxicities, it is important to underline that, within our study, a single case of grade 4 toxicity was observed, concerning a patient undergoing immunotherapy treatment with pembrolizumab. The symptoms, compatible with a manifestation of Erythema multiforme major, arose 7 days after the first infusion of the drug and rapidly worsened, resulting in a Grade 4 toxicity (desquamative-erosive lesions extending to >30% of the body surface).

A timely integrated management, based on the immediate withdrawal of pembrolizumab therapy and the administration of systemic intravenous corticosteroids, led to a gradual resolution of the condition over approximately 6 weeks.

Given the severity of the condition, in accordance with the guidelines, the immunotherapy treatment was kept permanently suspended.

### 3.3. Management of irAEs

In our cohort, the cases of toxicity that made it necessary to start systemic steroid therapy were 39%, i.e., the moderate and severe degrees.

In cases of mild toxicity, the administration of topical steroid therapy (in the form of ointment, mouthwash, or eye drops, depending on the site of the toxicity; 17%) or symptomatic (mostly represented by an oral antihistamine, 15%) was sufficient.

However, it is important to note that, despite a relatively high incidence of cutaneous irAEs, 28% of them underwent spontaneous resolution, without requiring any specific therapy.

In our study, the suspension of therapy with ICIs was mandatory in 22% of cases; of these, 7.7% required definitive discontinuation of immunotherapy.

This data, compared to a percentage of moderate–severe severity of 43%, confirms the importance of multidisciplinary, tempestive management of skin irAEs.

### 3.4. Prognosis of Patients with irAEs

Finally, we looked for the presence of a possible correlation between the appearance of dermatological irAEs and the OS of the patients.

**Figure 6:**
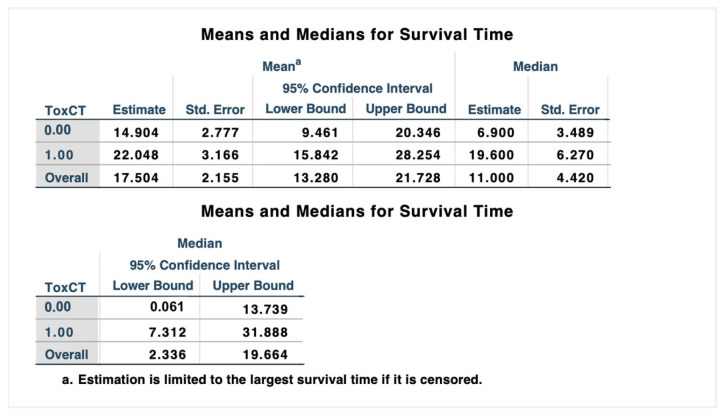
Means and medians for survival time in our study population.

**Figure 7:**
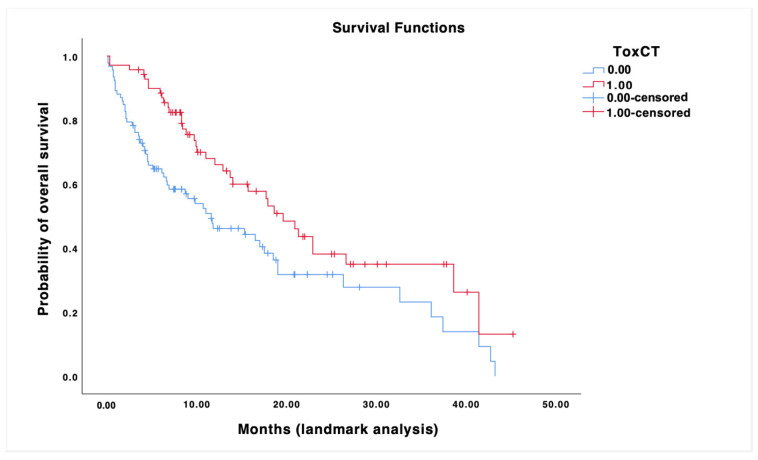
Survival curves of our cohort, displaying patients treated with ICIs for cutaneous melanoma who had been subject to skin irAEs within the first 3 months of therapy vs. patients who had not been subject to it.

A Landmark analysis was carried out 3 months after the onset of ICIs, including only patients who were alive at that point. These patients were then divided into two groups: patients who had been subject to skin irAEs within the first 3 months of therapy vs. patients who had not been subject to it. From the Kaplan–Meier analysis, it emerged that the appearance of skin toxicity was statistically significantly associated with the OS period, with a median of 6.9 weeks (95% CI 0.06, 13.7) in unaffected patients from skin toxicity and 19.6 weeks (95% CI 7.3, 31.9) in patients subject to one or more episodes of skin toxicity (Figure 6 and Figure 7).

## 4. Discussion

In the analyzed cohort of patients, considering the observational and prospective nature of this study and the sample size, the characteristics of the dermatological irAEs observed were generally consistent with those reported in major clinical trials [29,30,31]. Specifically, a cutaneous irAE incidence of 30–50% was confirmed, placing these toxicities among the most frequently encountered during treatment with ICIs. These manifestations predominantly affected patients treated with ipilimumab (48%) compared to those treated with PD-1/PD-L1 inhibitors (31–37%).

In line with the data in the literature, the most observed cutaneous irAEs were pruritus (29%) and erythematous rash (28%). However, certain irAEs occurred at a higher-than-expected incidence; mucositis (affecting the conjunctiva, oral mucosa, or vaginal mucosa), lichenoid dermatitis, and psoriasis (flare-up or first occurrence) each reported in 7% of cases. This finding should not be underestimated, especially considering that, in the case of the latter two conditions, the severity was classified as Grade 2 or 3.

Cutaneous irAEs appeared after an interval of 10 weeks, with a range varying from a few days to nearly 2 years from treatment start. This is consistent with findings from the main clinical studies conducted to date. The possibility of such prolonged latency intervals implies that patients who achieve significant and long-lasting responses to ICIs should not be considered exempt from the potential development of irAEs; on the contrary, it is appropriate that these patients undergo dermatological long-term monitoring [32].

The severity of cutaneous irAEs in our patient cohort was consistent with data previously reported in the literature: toxicities were predominantly mild (Grade 1) or moderate (Grade 2), at 57% and 36%, respectively, with a percentage of severe toxicity (Grade 3 or 4, requiring temporary or permanent discontinuation of immunotherapy) of 7%. In our study, only one case of Grade 4 toxicity was observed.

Cutaneous toxicities were managed through an integrated, multidisciplinary approach, where patients underwent specialized dermatological consultations at the first occurrence of symptoms or signs potentially related to ICI-induced cutaneous toxicity [33,34,35].

In accordance with guideline recommendations, mild irAEs were treated with symptomatic medications alone or, in some cases, with topical corticosteroids; moreover, approximately two-thirds of these resolved spontaneously [10]. This finding is reassuring, indicating that while the incidence of dermatological irAEs is high, these toxicities are often self-limiting and resolve without the need for specific treatments. Moderate-to-severe irAEs, on the other hand, required systemic immunosuppressive therapy in 39% of cases. Treatment discontinuation was necessary in 22% of cases (with 7.7% representing permanent discontinuations). This confirms that timely and integrated management of cutaneous irAEs positively influences the prevention of their progression and the consequent need to discontinue immunotherapy.

The investigation of potential correlations between cutaneous irAEs and patients’ medical histories, aimed at identifying possible predictive factors for the development of toxicity in patients treated with ICIs, confirmed that cutaneous irAEs are independent of age, sex, and the patient’s performance status.

We observed a tendency for different non-dermatological irAEs to present in association with dermatological toxicities in the same patient, either concurrently or subsequently. This observation, which has also been noted in previous case reports, is likely related to the generalized increase in immune system activity, particularly T-cell-mediated immunity induced by ICIs. Finally, we observed that the development of cutaneous toxicity is associated with a longer OS. This correlation between the appearance of specific dermatological irAEs (specifically rash, pruritus, and vitiligo) and a favorable prognostic outcome had already been described in patients treated with PD-1 inhibitors, particularly in the case of vitiligo in patients with advanced melanoma [36,37,38,39,40]. This phenomenon may be attributed to the fact that the development of such adverse effects could serve as a surrogate marker of treatment response, offering a reduced risk of disease progression and mortality compared to the absence of dermatological toxicities.

## 5. Limitations

Several limitations of this study need to be considered. First, our data are from a single center, potentially limiting the generalizability of our results. Moreover, the small sample size limits a thorough evaluation of other possible variables and considerations and/or hypotheses about the incidence of cutaneous irAEs in patients treated with different ICIs.

## 6. Conclusions

Despite the limitations related to the small sample size, we observed an incidence of cutaneous irAEs from ICIs in our cohort that was consistent with the data reported in the literature. Specifically, as previously described, a cutaneous irAE incidence of 30–50% was confirmed, placing these toxicities among the most frequently encountered during treatment with ICIs [29,30,31]. The most frequently observed cutaneous irAEs were pruritus and erythematous rash, and, in most cases, the toxicities were of mild-to-moderate severity.

Severe toxicities were extremely limited and, thanks to an integrated multidisciplinary management approach, were promptly identified and treated. This allowed for a reduction in the number of cases where discontinuation of ICI therapy was necessary, highlighting the importance of implementing a monitoring system for early diagnosis and management of these toxicities to further reduce the need for permanent treatment discontinuation.

Finally, we observed a better prognostic outcome in patients affected by cutaneous irAEs compared to those without such toxicities. Further investigation into this aspect could help definitively establish a role for cutaneous irAEs as surrogate markers of response to ICI treatment.

## Data Availability

No new data were created or analyzed in this study. Data sharing is not applicable to this article.

## Supplementary Materials

The following supporting information can be downloaded at: https://www.mdpi.com/article/10.3390/cancers16213679/s1, Table S1: Epidemiological data of the study population.

## Abbreviations

Cutaneous melanoma (CM); immune checkpoint inhibitors (ICIs); immune-related adverse events (irAEs); adverse events (AEs); overall survival (OS); Common Terminology Criteria for Adverse Events (CTCAE).
