# Supplementary material for: Cutaneous Toxicities of Advanced Treatment for Cutaneous Melanoma: A Prospective Study from a Single-Center Institution
Source: Cancers (Basel). 2024 Oct 30;16(21):3679. doi: 10.3390/cancers16213679 (PMC11545238; doi:10.3390/cancers16213679)
Supplement: Supplementary file 1 [file cancers-16-03679-s001.zip › cancers-3264910-supplementary.pdf]

Supplementary table

Table S1. Epidemiological data of the study population.

**STUDY COHORT: Patient under treatment with ICIs who developed cutaneous irAEs**	
Age	m = 71 (42; 88)
Sex	M = 45 (60)<br>F = 30 (40)
PS	m = 1 (0; 2)
Stage according to AJCC 2018	
II (B,C)	9 (12)
III	49 (65.3)
IV	17 (22.7)
ICIs	
- Ipilimumab	64 (48.1)
- Nivolumab	49 (37)
- Pembrolizumab	42 (31.4)
